# Is the Weight Over? An Improved HFpEF Risk Score

**DOI:** 10.1016/j.jacadv.2024.101037

**Published:** 2024-06-07

**Authors:** Mark N. Belkin, Ryan Sachar, Parag Goyal

**Affiliations:** a Section of Cardiology, University of Chicago, Chicago, Illinois, USA; b Department of Medicine, University of Chicago, Chicago, Illinois, USA; c Department of Medicine, Weill Cornell Medicine, New York, New York, USA

**Keywords:** dyspnea, heart failure, HFpEF

**Figure:**
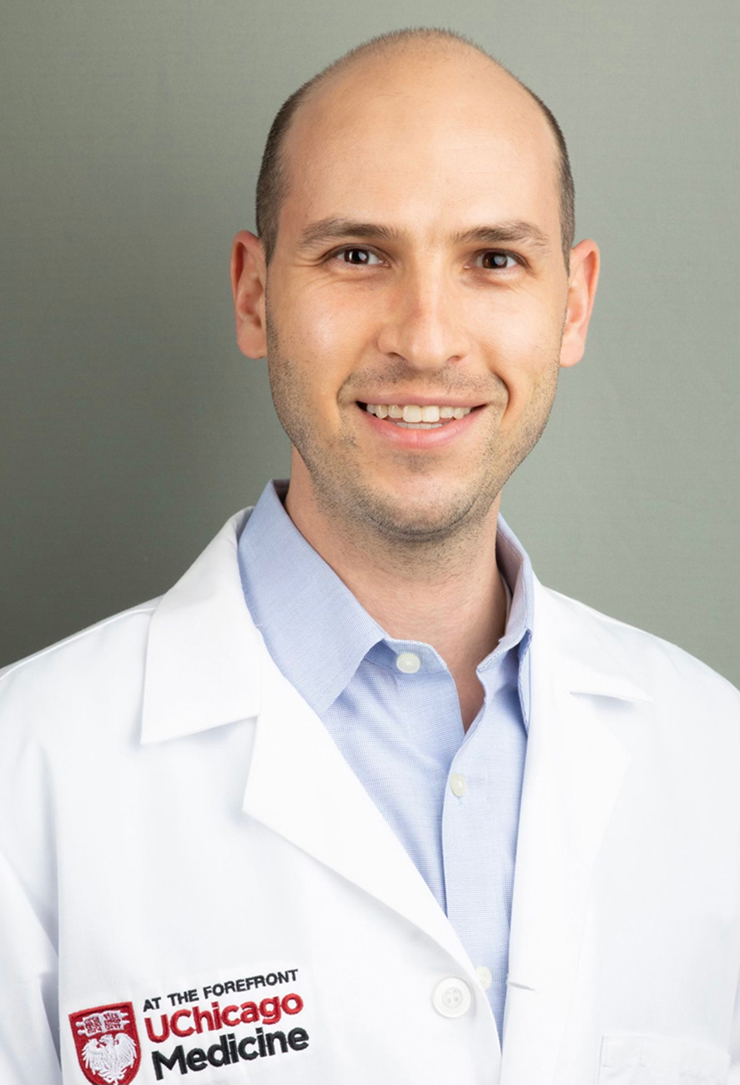

Given recent advances in therapeutic options for heart failure with preserved ejection fraction (HFpEF), noninvasive screening and diagnostic tools are critical to accurately identify patients and ensure they get timely treatment to improve health and prevent adverse outcomes.^1^ This can be challenging since patients with HFpEF often have other concomitant comorbid conditions that can present with similar symptoms—for example, obesity, present in over half of patients with HFpEF, can contribute to exercise intolerance and fatigue independent of HFpEF.^2^

There are 2 commonly used risk scores currently used for HFpEF in clinical practice. The H2FPEF score, published in 2018, is comprised of 6 factors: body mass index (BMI), age, number of antihypertensive medications, diagnosis of atrial fibrillation, echocardiographic evidence of diastolic dysfunction via E/e’ ratio, and echocardiographic measures of estimated systolic pulmonary artery pressure.^3^ The HFA-PEFF score, introduced in 2019, includes additional echocardiographic data points, as well as natriuretic peptide levels.^4^ Despite the high specificity and sensitivity of these scores individually, in a validation study of patients with unexplained dyspnea, the H2FPEF and HFA-PEFF scores resulted as “indeterminate” in approximately half of patients. In current HFpEF algorithms, indeterminate scores result in downstream recommendations for exercise invasive hemodynamic, or stress echocardiographic, testing to confirm diagnosis; however, neither of these are routinely done in real-world practice.^5^ While these tools can be helpful, the aforementioned limitations indicate the need for improved tools for addressing diagnostic uncertainty in practice.

To create an improved predictive score for HFpEF, Bermea et al combined 2 established cohorts of patients with HFpEF and elevated BMI from their respective institutions.^6^ They performed a retrospective analysis on 309 patients with HFpEF and 134 controls without any cardiovascular disease or echocardiographic abnormalities. The team identified 20 salient clinical and echocardiographic variables and applied a Gradient Boosting Machine to rank these variables’ association with the diagnosis of HFpEF. The 4 variables most predictive of HFpEF, in order, were: 1) BMI; 2) estimated glomerular filtration rate; 3) left ventricular mass indexed; and 4) left atrial: left ventricular ratio (LA/LVr). They then built a multivariable logistic regression model using these variables to create the novel HFpEF-JH score. This resulted in a sensitivity of 0.83, a specificity of 0.82, and an area under the curve (AUC) of 0.88 for the diagnosis of HFpEF with a cutoff of 0.83 (scale 0-1). Importantly, the HFpEF-JH score had improved sensitivity and AUC compared to the H2FPEF score (sensitivity of 0.19 and AUC of 0.74 for a cutoff of 6 on a scale of 0-9). While the HFA-PEFF score was not included in this analysis, head-to-head comparison of the H2FPEF and HFA-PEFF scores indicated lower AUC and sensitivity for the latter in patients with unexplained dyspnea.^5^

In further comparing these 2 scores, it is notable that the HFpEF-JH score performed significantly better than the H2FPEF score in patients with BMI >30 kg/m^2^ (*P* < 0.001). It is frequently difficult to discriminate the cause of dyspnea in the growing population of patients with obesity. Accurately making a diagnosis in this population is key both to identify the appropriate patients, and ideally avoid the diagnostic uncertainty associated with the indeterminate range scores frequently seen with the H2FPEF and HFA-PEFF scores.^5^ The improved discrimination in the population with BMI >30 kg/m^2^ is welcome and has the opportunity to improve the evaluation of dyspnea in this population.

The HFpEF-JH score may initially be interpreted as too intricate, both with its incorporation of echocardiographic parameters that are not routinely assessed and its complex calculation. However, the authors do provide an online tool for clinical use of their novel score. Additionally, they note that the HFpEF-JH may be a refinement of the H2FPEF score given that estimated glomerular filtration rate reflects age, LA/LVr may reflect the presence of atrial fibrillation, and left ventricular mass indexed may be related to the number of antihypertensives. One could also consider this an integration of the clinical and echocardiographic aspects the current validated scores—with BMI from H2FPEF and incorporation of left atrial and ventricular structural measures from the HFA-PEFF score. Lastly, it is important to note that the 4 variables identified by the machine learning algorithm have each individually been shown to be associated with more advanced HFpEF disease, including a recent study correlating LA/LVr with exercise peak oxygen consumption and diastolic dysfunction in patients with HFpEF.^7^, ^8^, ^9^, ^10^ Considering these data, along with the impressive improvement in AUC and sensitivity shown by the authors, the HFpEF-JH score is a needed advancement to the field.

The novel HFpEF-JH score shows promise, but, as the authors point out, it warrants confirmation with regard to external validity, and in comparison to non-healthy controls with unexplained dyspnea. Furthermore, additional validation against invasive hemodynamic diagnosis of HFpEF would further solidify the reliability of this score, as has been done with the H2FPEF score.^5^^,^^11^ It is promising that the authors redemonstrated a diagnostic sensitivity of 0.83 in a sensitivity analysis of a subset of patients with invasive hemodynamics, but it is not evident how many individuals of the cohort this included.

The authors should be congratulated on developing a sensitive HFpEF-JH score that can better identify patients at risk for HFpEF, potentially reducing the risk for underdiagnosis or misdiagnosis of this condition, particularly in patients with obesity. Continually improving the diagnostic armamentarium will not only help better identify patients with HFpEF so that they can receive the appropriate treatment but also has the potential to improve conduct of clinical trials which may be excluding a disproportionate number of patients with obesity.^12^

## Funding support and author disclosures

The authors have reported that they have no relationships relevant to the contents of this paper to disclose.
